# A qualitative investigation of experiences of care and illness perceptions related to self-management behaviors in chronic kidney disease

**DOI:** 10.1080/21642850.2026.2699481

**Published:** 2026-07-08

**Authors:** Malin Ekholm, Henna Hämäläinen, Virpi Rauta, Keegan Knittle

**Affiliations:** a Faculty of Sport and Health Sciences, University of Jyväskylä, Jyväskylä, Finland; b Department of Nephrology, Helsinki University Central Hospital, Helsinki, Finland; c Discipline of Social Psychology, Faculty of Social Sciences, University of Helsinki, Helsinki, Finland; d Strategy and Development, Helsinki University Central Hospital, Helsinki, Finland

**Keywords:** Chronic kidney disease, self-management, illness perceptions, patient experiences, qualitative research

## Abstract

**Background:**

Chronic kidney disease (CKD) requires active self-management to slow progression, yet adherence is often low. To inform intervention development, this qualitative study explored CKD patients' illness perceptions and care experiences, and examined how these factors may interact to shape self-management.

**Methods:**

Eighteen adults with CKD stages 2–5 were recruited from a nephrology clinic and interviewed immediately after routine care encounters. Semi-structured interviews were analyzed using both inductive and deductive reflexive thematic analysis (informed by the Common-Sense Model of Self-Regulation).

**Results:**

Patients generally perceived CKD as chronic but controllable, with few noticeable symptoms and limited immediate consequences. Low symptom burden often reduced the perceived importance of self-management in daily life, whereas understanding the reasons for treatment and lifestyle changes increased motivation. Patients described treatment and control beliefs as moderate, but patients struggled to perceive the effects of dietary changes. Access to clear information, continuity of care, and trust in healthcare and practitioners were highlighted as important for coherence and confidence in self-management. Emotional responses were often alleviated through increased knowledge and supportive interactions with healthcare staff.

**Conclusion:**

Mapping experiences to illness perceptions revealed multiple pathways through which care processes may support or hinder CKD self-management.

## Introduction

Chronic kidney disease (CKD) is a costly and increasingly prevalent condition marked by a gradual loss of kidney function (Jha et al., 2023). As CKD progresses, kidney function declines, eventually necessitating dialysis treatment (Stevens et al., 2024). Prior to dialysis, evidence suggests that several self-management behaviors can slow disease progression. These include, for example, taking medications to control blood pressure, adhering to a kidney friendly diet (i.e. avoiding sodium, potassium, and phosphorus), and avoiding excess fluid intake (Ikizler et al., 2020; Stevens et al., 2024).

Self-management demands evolve as the disease progresses. In earlier stages (2 and 3), the focus is on managing the underlying condition (often hypertension or diabetes) and slowing kidney function decline through medication and lifestyle changes such as diet. As CKD advances, dietary modifications become more substantial and medication regimens grow. By stage 5, strict fluid and dietary restrictions accompany preparation for kidney replacement therapy or conservative care (Ikizler et al., 2020; Stevens et al., 2024).

Despite evidence of the importance of these self-management behaviors, adherence is typically low (e.g. Tesfaye et al., 2021; Vr & Kang, 2022). While numerous interventions have aimed to increase treatment adherence and self-management in CKD, research on such interventions has mostly centered on patients who are already undergoing dialysis, and the effects of these interventions are mostly small (Ekholm et al., 2025). To plan for effective interventions, and develop CKD care pathways, intervention planners and health care practitioners (HCPs) must first understand the determinants of self-management behaviors (Eldredge et al., 2016). This includes the needs and experiences of patients living with CKD and how they navigate the healthcare system. Patient experiences play a major role, as they relate to both patient satisfaction, but also to health outcomes (Doyle et al., 2013).

While patient experiences are recognized as essential for health-care quality, clinical effectiveness and patient safety, there is not a clear consensus of the definition of the term (Oben, 2020). When patient experiences are conceptualized in relation to interaction with healthcare, the term could be defined as ‘*the sum of all interactions, shaped by an organization's culture, that influence patient perceptions across the continuum of care’*; including personal interactions with HCPs, organizational culture at the unit of care, patient perceptions, and the healthcare journey of the patient (Wolf et al., 2014). This also relates to recent KDIGO guidelines for creating data-driven collaborative care in CKD (Li et al., 2025). While this definition does broach on patient perceptions, it does not adequately address patient experiences or perceptions of the illness or condition which is being treated. These perceptions play an important role in various health behaviors, which makes them an important domain of patient experiences (Hagger et al., 2017; Leventhal et al., 2016). In this study we will thereby define patient experiences not only as experiences rooted in the interactions with healthcare systems, but also the experiences of living with CKD, and what illness perceptions patients have of CKD.

According to the Common-Sense Model of Self-Regulation (CSM; Leventhal et al., 2016), people construct cognitive and emotional representations of their illness (or illness perceptions) based on various situational stimuli (e.g. symptoms, lay information, or information from expert sources). These illness perceptions are constructed around six different dimensions: illness identity, causal attributions, illness timeline, perceived consequences, perceived control over illness, and coherence. Identity refers to the symptoms a person attributes to the illness. Causal attributions concern beliefs about what brought the illness on. Timeline captures beliefs about how long the illness will last (acute versus chronic) and whether symptoms come and go over time. Consequences cover how a person perceives the illness to affect their life, for example financially, physically, or socially. Control reflects both the personal control a person has over their illness (e.g. through self-management) and the extent to which they perceive that treatment manages it. Coherence refers to how well a person feels they understand their illness. In addition to these cognitive representations, the CSM also includes the emotional responses people have because of an illness or health threat (Hagger et al., 2017; Leventhal et al., 2016; Moss-Morris et al., 2002).

Illness perceptions provide a useful framework for examining patient experiences, and are linked to a range of health outcomes and coping behaviors (Hagger et al., 2017). In CKD, they predict mortality (Muscat et al., 2020), disease progression (Meuleman et al., 2015), treatment adherence and self-management (Chilcot et al., 2010; O'Connor et al., 2008; Velez-Velez & Bosch, 2016) depression (Chilcot et al., 2013) coping styles, and quality of life (Knowles et al., 2023). Illness perceptions are also modifiable, and interventions are most effective when delivered before perceptions become firmly established (Clarke et al., 2016).

In non-dialysis dependent CKD, illness perceptions have been linked to mortality and decline in kidney function (Meuleman et al., 2015; Muscat et al., 2020), and to psychosocial outcomes like distress (Muscat et al., 2021). Qualitative work in this group has identified illness perceptions related to the seriousness of CKD (illness identity, consequences, emotional responses, and concerns) and to its manageability (illness coherence, personal control, and treatment control). Over time, patients tend to develop more unhelpful seriousness-related perceptions but more helpful manageability-related perceptions, and several modifiable perceptions do not improve through nephrology care alone (Meuleman et al., 2024). How these perceptions interact with patients' experiences of care to shape self-management, however, remains less well understood.

This research thereby aims to examine CKD patients' illness perceptions and experiences of care, and to identify how interactions between these factors might be leveraged within CKD treatment to promote self-management behaviors. The study aimed to examine the experiences across the early stages of the CKD patient journey, from the first nephrology visit through to follow-up visits after a dialysis modality has been chosen but before dialysis or dialysis training has begun.

## Materials and methods

The Research Ethics Committee of the Helsinki University Hospital granted ethical approval for this qualitative interview study on August 9th 2023. All study procedures were conducted according to the Declaration of Helsinki, and study data were collected between November 2023 and May 2024.

## Participants and recruitment

Purposive sampling was used to recruit a diverse sample of patients in different stages of CKD, different illness profiles (e.g. comorbidities), various educational backgrounds and age. Eligibility criteria for patients were: ≥18 years old, patient had received a referral to the nephrology clinic due to diagnosed or suspected CKD, and sufficient understanding of the Finnish language. We aimed to recruit patients who had not yet started dialysis training or begun their chosen dialysis treatment. Exclusion criteria were patients with cognitive impairments or active substance abuse. Patients with cognitive impairment were excluded because the interview required reflection on past care encounters and engagement with abstract questions about illness perceptions. Patients with active substance use disorder were excluded because this group faces complex and overlapping care needs (Wu et al., 2018), and reduced access to kidney transplantation (Sandhu et al., 2011), which shapes their healthcare experiences and self-management trajectories in ways that fall outside the focus of this study on CKD-specific care.

As participants were to be interviewed after encounters with HCPs, the research nurse screened and identified potential participants from upcoming scheduled appointments. Potential participants were contacted via telephone. The research nurse provided potential participants with basic information about the study, and provided interested patients with a participant information sheet. Patients who agreed to participate were sent a consent form via email, which they signed and returned to the research nurse. Participants were finally told that they may ask any questions or withdraw from the study without stating a reason.

Recruitment was halted after 18 participants had been enrolled. This sample size is comparable to other qualitative studies of patient experiences in CKD (e.g. Meuleman et al., 2024; Sledge et al., 2023), and provides sufficient information power through a focused study aim, use of an established theoretical framework, and in-depth interviews (Malterud et al., 2016). The study sample comprised of 61% male participants and 39% female participants, with men slightly overrepresentedcompared to population level prevalence rates (Vosters et al., 2024). The median age of participants was 62.5 years (IQR = 50.5, 71.8). Renal function, as measured by estimated glomerular filtration rate (eGFR), ranged from 7 mL/min/1.73 m^2^ to 79 mL/min/1.73 m^2^ with a median of 15 mL/min/1.73 m^2^. In terms of stage of CKD, one patient (5.56%) was in stage two, three patients (16.67%) were in stage three, five patients (27.78%) were in stage four, and nine patients (50.00%) were in stage five at the time of recruitment, one of which had acutely begun dialysis treatment prior to participation in the interview.

Recruited participants attended a variety of encounters with healthcare practitioners prior to their interviews. Four participants attended their first appointment with a nephrologist at the nephrology unit, two participants had an appointment to a dietitian, five participants attended a dialysis decision clinic with a nephrologist and a nurse, two participants attended group patient education sessions, and five participants, who had already chosen a dialysis modality, attended follow-up appointments with a nephrologist. Patients are referred to the nephrology unit for suspected or confirmed kidney disease, such as significant proteinuria, unexplained or rapidly rising creatinine, or severe kidney failure requiring treatment planning. The first visit serves to confirm or refine the diagnosis through medical history review, laboratory testing, and where indicated, kidney biopsy. Including patients still in the diagnostic phase made it possible to capture illness perceptions and care experiences from the earliest stages. Appendix I shows a simplified version of the typical patient journey in the study context. The timing of some encounters may vary between patients in practice, and patients may have several other visits relating to comorbidities in other units.

Individual-level demographic data are not presented to ensure patient anonymity and confidentiality, particularly given the small sample size and clinicians being aware of study participation, which could potentially allow for identification of participants.

## Data collection

Data was collected via semi-structured individual interviews, conducted face-to-face immediately following an encounter with a healthcare practitioner in a nephrology unit in the Helsinki University Hospital. As experiences may be shaped and re-interpreted by later reflection or external influences, it is important to conduct interviews as soon as possible after encounters (e.g. Smith et al., 2009; van Manen, 1997). Participants were however asked prior to each interview whether they had sufficient time and energy to participate. In one case, the interview was conducted by telephone a few days later, as the participant reported feeling fatigued following the encounter.

Prior to conducting interviews, an interview protocol was developed based on previous literature and research about self-management in CKD, patient experiences, and illness perceptions (Ekholm et al., 2025; Leventhal et al., 2016; Meuleman et al., 2024; Moss-Morris et al., 2002). The interview protocol covered patients' illness backgrounds, experiences with CKD, treatment and self-management, and their experiences with the care they had received. Self-management was addressed through open-ended questions tied to the Common-Sense Model, for example through perceived consequences (e.g. ‘How would you describe what CKD is and how it affects your life?’ and ‘Are there things you need to do or be mindful of because of CKD?’), rather than by asking directly about specific behaviors. This design reduced the risk of leading patients toward particular actions and allowed them to describe self-management in their own words. When patients raised specific behaviors, these were probed with open-ended follow-up questions*.* The interview protocol was piloted with four patients who had just been referred to the nephrology clinic, and were attending their first visit with a nephrologist. After piloting, the protocol remained largely the same, but a think-aloud assignment was added to the end of the interview, in which patients were presented with questions about illness perceptions (e.g. ‘*Coherence—how well do I understand my illness?’*) and asked to think aloud. This interview support was developed based on a previous study exploring illness perceptions in CKD (Meuleman et al., 2024). For a complete interview protocol, translated from Finnish to English, see Appendix II.

Interviews ranged from 17 to 72 min in length, with a mean duration of 39 min (SD = 15). The shortest interviews were those conducted with patients during piloting, after their first nephrology visit. The interviews were conducted by a doctoral researcher in social psychology (ME) with experience in conducting qualitative research interviews and prior familiarity with CKD literature and the Common-Sense Model. The interviewer had no prior relationship with the patients. Interviews were audio-recorded using two digital voice recorders (OM System WS-882) provided by the primary researchers institution. Participant characteristics were extracted from hospital registers by the study nurse.

## Data analysis

Interview data were analyzed using reflexive thematic analysis, in which the researcher takes an active role in constructing and interpreting themes in the data (Braun & Clarke, 2006, 2019, 2021). In this study, we assumed that there is depth and complexity in the experiences of patients, which is best examined inductively. We also assumed that patients have some kind of illness perceptions, and we aim to examine how these are described and how they may relate to self-management behaviors. This study therefore used both inductive and deductive reflexive thematic analysis to identify themes, to provide structure, and to link findings to existing knowledge and theoretical frameworks. Surfacing these assumptions, alongside the primary researcher's prior experience, was treated as part of ongoing reflexive practice throughout the analysis.

An inductive approach was taken to gain a comprehensive understanding of patient experiences of care, while a deductive approach was taken to explore illness perceptions related to self-management behaviors. This hybrid inductive and deductive approach has previously been proven useful in examining patient experiences in CKD, and in other settings (Fereday & Muir-Cochrane, 2006; Meuleman et al., 2024; Proudfoot, 2023). As a final step, our analyses also applied matrix analysis (from framework analysis methodology) to systematically analyze the intersections of inductive and deductive themes in the context of CKD self-management behaviors (Klingberg et al., 2024).

The thematic analysis followed Clarke and Braun's 6-step approach to thematic analysis (Braun & Clarke, 2021), which started with familiarization with the data set. This included transcribing the interviews verbatim and reading and re-reading the transcripts. Transcription is considered a key phase in qualitative analysis (Bird, 2005) and was thereby done by the primary researcher (ME). After familiarization, the data was analyzed and coded for descriptions of patient experiences, self-management behaviors, and experiences of living with CKD (Step 2 generating initial codes) by one researcher (ME). Coding was done in ATLAS.ti (version 24.1.1; ATLAS.ti Scientific Software Development GmbH, 2024). From the coded data, potential themes were identified (step 3 searching for themes) in two different ways: inductively, by identifying themes related to patient experiences and self-management, and deductively, by organizing codes according to CSM principles (Leventhal et al., 2016; Moss-Morris et al., 2002). Initial codes were reviewed and discussed by two researchers (ME, KK) to ensure that the themes consisted of coherent extracts, and that the themes adequately represented the data set. At this stage some themes were merged or discarded, and additional missing themes were identified (step 4 reviewing themes). Themes and codes were reviewed and critically discussed throughout the analysis process by two researchers (ME, KK), and identified themes were discussed with a multidisciplinary team of healthcare practitioners, and researchers in the field of patient experience and CKD (including HH, VR, and other experts). Once themes had been reviewed, they were clearly labelled and defined (step 5 defining and naming themes). Results were finally presented, and illustrative quotes were selected and translated from Finnish to English by a bilingual researcher.

Once themes had been defined and labelled, a matrix was constructed following framework analysis methodology (Klingberg et al., 2024), with the inductively identified themes relating to care experiences as rows, and the deductively coded illness perceptions dimensions as columns. The relationships between care experiences and illness perceptions were identified primarily by examining overlapping codes in ATLAS.ti, supported by thematic mapping on paper. For each intersection, coded data in which care experience themes, illness perceptions, and self-management co-occurred were extracted, and illustrative quotes were selected for each cell to capture the relationship between themes. The resulting matrix is presented in Appendix III.

Several of these analysis steps, including discussion of codes and themes, piloting interviews, open-ended questions, and the combination of inductive and deductive approaches, also serve to support analytic rigor in line with the trustworthiness criteria proposed by Lincoln and Guba (1985): credibility, transferability, dependability, confirmability, and reflexivity.

## Results

### Illness perceptions and self-management

Most patients described experiencing few symptoms of CKD (i.e. low illness identity), perceived relatively few consequences of CKD, described moderate treatment and personal control beliefs, perceived CKD mostly as chronic, and described a variety of emotional responses related to diagnosis, the thought of being sick, and upcoming dialysis treatment. Of these illness perceptions, identity, consequences, control beliefs and coherence appeared central to self-management behaviors.

#### Identity and consequences

Some participants in this study attributed symptoms of fatigue, swelling, itching skin, and lack of energy to their CKD. While some patients described experiencing strong fatigue and frequent lack of energy, symptoms were mostly described as mild. Some even described that they would not know that they had CKD if it was not for the healthcare staff informing them about it.

*I just asked the doctor about how I will know that I am sick, because I don't feel sick. Always felt normal.* P12

The lack of (severe and frequent) symptoms meant that, for some participants, self-management behaviors were not at the front of their minds. CKD was described as something absent from everyday life, and thereby patients might not pay attention to self-management.

*…when I come to [the hospital] I am reminded [about self-management]. Because like I said, so far this disease has been like in the background and has not had that much of an effect. You have to sort of like be more aware and disciplined.* P17

The perceived immediate consequences of CKD centered primarily on frequent care visits (e.g. laboratory testing and follow-up), medication taking, and dietary modifications or restrictions. Some patients also described social, occupational and behavioral consequences arising from symptoms.

*in a way it has had an impact on my work. And then this exhaustion, so you don't have the energy to do things like you used to. Hobbies, social life. Because the exhaustion is, it is quite strong.* P11

When looking at the long-term impacts of CKD participants had more concerns about potential future consequences, including how their CKD might progress and how starting dialysis might negatively impact their lives.

*[CKD] has not been a huge like thing, but now of course we are discussing this dialysis treatment, so it will have an impact, and that is probably the biggest question.* P17

Not feeling sick or not experiencing serious consequences of CKD may also result in patients feeling reluctant to accept treatment or consider dialysis modality options..

*When you are not feeling that sick it feels quite—you do not readily want to sleep next to that kind of a [dialysis] machine.* P18

#### Control beliefs

Both personal control and treatment control beliefs were strong for most patients. Patients described having control over their health status through taking medication, diet, exercise, avoiding stress, and an overall healthy lifestyle. Some patients also recognized that there are limits to what they can do on their own, and that collaboration with their healthcare practitioners is important.

*I can [control my health and illness] a lot. I could make things go to shit if I wanted to, If I wanted to make things worse or if I didn't take care. Like I do have a lot of control, but let's say that I cannot make myself healthy on my own, like I need these healthcare professionals to take care of that part.* P12

While control beliefs were quite strong, many patients did not have a clear perception of how their self-management behaviors impacted their condition. Although some patients described feeling better as a result of making changes to their diets, most did not perceive that dietary changes led to differences in their health or laboratory test results. By contrast, patients reported that the effects of medications were easier to perceive than those of dietary changes. The invisibility of these effects was described by some patients as impacting both personal control beliefs and motivation to self-manage.

*But for diet not so much, like it does not have direct like, or you can't say that doing something would make you feel worse. That also makes being disciplined hard. You do not get immediate feedback that you should not have done that [laughs].* P17

#### Coherence

Many patients described understanding their CKD mostly through changes in laboratory values. Their understanding of normal ranges for different biochemical parameters was based on information provided by HCPs and digital platforms that allowed patients to self-monitor their laboratory test results. While knowledge about normal parameters of laboratory results helped patients understand their current conditions, the results themselves were described as somewhat abstract.

*There have been a lot of different lab values. But they remain these quite abstract numbers. Like it should be some number, and now it is a much bigger number or the number is too small. Or the value is something that I don't quite understand. I have sort of mechanically been able to monitor them, but not really understand what all of these things are.* P17

Coherence also emerged as important in terms of treatment. Not all patients were sure about how treatment works and what different medications do. When patients gained more understanding of CKD and its treatment (high coherence), they were also more motivated to self-manage.

*Well in the beginning yes when I was in the dark about things I was worried. But now that I've figured a lot of things out it is not that bad. And I think that might be what maintains the motivation to be healthy. Because you know there is a really important reason for it.* P4

#### Timeline perceptions

Most patients described CKD as being chronic, such that they would likely need to take medication and think about their diet for the rest of their lives. Some, however, described CKD as more cyclical in nature, having phases, such as dialysis and a kidney transplant. Kidney transplant was described as a kind of cure for CKD, despite patients acknowledging that they would be required to take immunosuppressants.

*Well right now it is chronic, but if I get a working kidney, then. Ehm, then I can say that it was temporary. Like I will never get rid of for example the immunosuppressants […] I think in a way that my current kidney is sick, but if I get a new one it is healthy.* P12

Despite perceiving CKD as chronic, most patients described that it can be controlled through self-management. One patient, who did not perceive CKD as chronic, described the condition as something they still hoped to cure or reverse with active self-management.

*I think that it is chronic. Of course, with diet and exercise—with diet we can control this. In the sense that [the kidney values] would remain stable. But the kidney disease, like the root cause of it will remain.* P6

*[treatment and self-managemet] has to be the reason that this is gradually getting better. And I hope that it will be completely cured, like ehm– it is not yet completely better.* P18

#### Emotional responses

Patients described various emotional responses related to time before diagnosis, being diagnosed, getting treatment, receiving more information about CKD, and thoughts of upcoming dialysis. Uncertainty related to getting a diagnosis or thinking about a future on dialysis caused worry, distress, depressive symptoms and fear for some, but patients describe that these reactions have to some extent calmed over time. Unexpected CKD diagnoses came as a shock to patients, but those with comorbidities such as diabetes reported less intense negative reactions, as they were accustomed to undergoing treatment or had even anticipated a CKD diagnosis. Among all patients, reactions however varied. Some said their diagnosis felt unfair and left them feeling sad or bitter about having to manage another illness. One patient, by contrast, experienced their diagnosis as a relief, since it explained their deteriorating health.

Patients did not describe a direct relationship between emotional reactions and specific self-management behaviors, but emotional reactions seemed to play a key role in coping overall. Some patients said that understanding more about their condition (coherence), treatment options, and things they can do to self-manage their CKD (control beliefs) had calmed their emotional reactions.

*At first I was shocked and afraid, but I got used to the thought quite fast. The more information you get, the more you in a way submit to it. Knowing there is treatment and that I won't die of this and that I have time to sort of prepare myself for treatment also calmed me down.* P12

### Patient experiences of care

Thematic analysis revealed six main themes related to patients' experiences of CKD care: diverse definitions of care, continuity of care, availability of information, active role of patient in care, trust in healthcare, and social support. The identified themes and descriptions are summarized in Table 1.

**Table 1.** Themes related to patient experiences of CKD care.

Theme	Description
Definitions of care	Patients differed in their perceptions of what care or treatment entails. Some defined care only as medication and dialysis, others defined care as including diet, exercise, and interactions with HCPs.
Continuity of care	Patients underlined the importance of consistent contact with HCPs, and that repeated interactions with the same HCPs were preferred to interacting with a changing group of HCPs. Coordinated and clear care provision was described as important throughout the patient journey.
Availability of information	Access to clear information was highly valued, as it helped patients understand their illness, their treatment options, and the choices available to them. While the abundance of information was viewed positively and as a sign of support, many patients noted that they needed time to process and absorb everything they were given.
Active role of patient	Patients felt they must take an active role in managing their CKD. This involved following medical advice, self-management, and making their own decisions about dialysis, as well as advocating for themselves within the healthcare system (e.g. pushing for care and referrals, facing delays in accessing specialists).
Trust in healthcare	Patients' experiences were grounded in trust in their healthcare practitioners, including confidence in their competence, empathy, and reliability, as well as trust that the guidance they received was accurate and relevant for self-management and choosing a dialysis modality.
Social support	Social support from family, friends, and peers was viewed as essential to well-being, but patients noted that finding relatable peer support could be difficult when peers differed in age, CKD stage, or life circumstances. Some also felt that seeking social or psychological support added to their burden, becoming yet another task alongside frequent CKD-related care.

Note: HCPs = healthcare practitioners; CKD = chronic kidney disease.

#### Definitions of care

Participants described diverse definitions of care; some perceiving ‘care’ only as medication and upcoming dialysis treatment, while others also included diet and interaction with HCPs as part of their care.

*I have not received any care, so [the effects of treatment] will ehm—that will become clear once I start receiving the first dialysis treatments.* P4

*If we think about this kidney disease then the care, well. Maybe this information from the dietitian of course is part of this care, self-care. So that you get the right information.* P6

#### Continuity of care

Many patients emphasized the importance of continuity of care, noting that consistent and coordinated interactions with HCPs built trust and reduced worry. Some preferred to see the same doctor, valuing a practitioner who knew them beyond what is written in their medical records. Frequent changes between doctors had the opposite effect, lowering trust and adding to worry.

*I have to answer the same questions and explain the same things again when the doctor is always new, and I get a lot of different answers or different perspectives when the doctors change. The nurses have been really nice. But change in doctors is—I have sometimes felt that as untrustworthy, or like it feels like I am not getting the best possible care when doctors change.* P11

#### Availability of information

Access to clear, reliable, and timely information was valued, as the availability of information enabled patients to understand their illness, how it is treated, and make choices regarding their treatment.

*I have been really happy and even surprised how great these information sessions have been, and that there really is so much information made available. It feels like there is a lot of like help available.* P17

*When it is time—or if there is a must to make a choice [of dialysis modality], then now [after the information sessions] you can think a little bit further as you know what the options are. Like before you know you are sort of panicked. But now you can think about [dialysis modalities] like options, rather than something bad you have to do.* P18

#### Active role of patient

Many patients acknowledged that they needed to take an active role in their care, which involved both self-management of CKD and shared decision-making about dialysis modality. Patients expressed that despite being in good care, they are responsible to follow recommendations made by HCPs, and that the choice of dialysis modality was or will ultimately be up to themselves. Patients also spoke of the need to advocate for themselves and push for care and referrals. Some recounted very negative experiences in primary care, including delayed referrals and having to demand to see a specialist.

*You have to understand and remember that–it does not work like–even if this care is excellent and you get a lot of information, everything is still up to how you use that information and what you do.* P17

*I told [the primary care doctor] that I am worried about my creatinine values rising[…]But the doctor just said that the values are just a little bit on the rise, like that's nothing. Well then it rose again, but still nothing. It was just a little bit elevated—if it is twice what it should be to be in normal range I don't think it’s normal. And then I asked, like could we possibly look into this a little bit, like what is wrong? Then it took a few months and then this primary care doctor referred me.* P8

#### Trust in healthcare

Patients spoke about trust in their healthcare practitioners, which rested on perceptions of competence, empathy, and reliability. They trusted the information HCPs gave them, valuing it over other sources such as the internet, and considered the advice they received on self-management and the choice of dialysis modality relevant to their situation.

*I would assume that I will start to feel better [after starting dialysis]. At least that is what [the HCPs] have been telling me. I will feel completely different. And I completely trust that. Otherwise, all this would be for nothing if you do not have trust.* P9

*I have no way of knowing if I would not restrict my diet, like I don’t know if this illness would have progressed or gotten worse. So when it comes to diet I can’t really say. So I have just hoped and liked lived according to the recommendations in good faith and believed that there is a point to it. And I’m sure there is. I doubt they would emphasize it if not.* P14

#### Social support

Patients valued social support from family, friends, peers, and patient organizations as a complement to professional care and an important part of overall well-being. They found talking with other patients (experts by experience) particularly valuable, but some struggled to find peer support they could relate to. The peer support provided by experts by experience and coordinated by the hospital or patient organizations was not always perceived as relatable e.g. due to differences in peer age, CKD stage, or socioeconomic status.

*I called the [patient organization] and they said that they could give me someone, or someone could call me. One person called me a few times—a woman probably my age, but like she was in such a different situation, like she had already received a transplant years ago and I was just starting this process. We were not quite on the same page so that did not continue.* P10

In relation to both the active role of the patient and social support, some patients also described finding social or psychological support as a burden, as it was yet another thing that they had to do, in addition to the care visits related to CKD and possible comorbidities.

*I’ve never really had any type of [social] support—I do remember that I’ve received some information about it. Like there are some organizations. But then the thing is that it is sort of another thing in addition to all this [other care].* P11

### Interactions of illness perceptions and care experiences

Examining the interactions between care experiences and illness perceptions revealed several connections, showing that care experiences can shape illness perceptions (or vice versa) and, in turn, influence (motivation for) self-management behaviors. The matrix with patient quotes illustrating these interactions are presented in Appendix III.

#### Identity and care experiences

Patients' care experiences shaped how they perceived the somatic components of CKD (e.g. illness identity). In particular, ensuring access to information seems crucial to helping patients correctly attribute their symptoms and to anticipating potential symptoms as CKD progresses. Continuity in care, including regular follow-up, may also serve as a reminder for treatment and self-management for patients who experience few symptoms. Lastly, social support, especially from peers with similar experiences, provides valuable insights into what symptoms are associated with CKD, and what to expect over time.

#### Consequences and care experiences

Continuity of care can in itself be perceived as a negative consequence of CKD, as patients are required to spend a lot of time at the hospital. Consistent follow-up may, however, also feel reassuring and reduce anxiety about both present and future consequences. The availability of information may shape how patients understand potential consequences, especially regarding upcoming dialysis, and this information can support informed decision-making and self-management.

The need for the patient to take an active role in care and self-management may be considered a consequence of CKD, which can be perceived as demanding. Trust in healthcare is critical, as skepticism arises when patients feel that HCPs minimize the seriousness of future consequences, particularly dialysis. Similarly, peer support (e.g. through peers providing information and sharing experiences) can help patients anticipate challenges, both relating to dialysis and CKD overall, but its effectiveness depends on relatability; when peers’ life situations are perceived as different, patients may doubt or dismiss the relevance of their experiences.

#### Control beliefs and care experiences

How patients define ‘care’ may affect whether they view medication adherence or dietary changes as meaningful for controlling CKD. Regular follow-ups and clear communication with healthcare practitioners, i.e. continuity of care, reinforce patients' understanding of how self-management and treatments influence their health. Availability of information, including explanations of laboratory results through digital platforms or appointments, further strengthens control beliefs by showing tangible links between self-care behaviors and health outcomes.

Self-monitoring (of e.g. laboratory values or blood pressure) can build patients' sense of control, and trust in HCPs seems to serve as a foundation for believing in the effectiveness of treatment and lifestyle changes—especially when patients do not perceive effects of their behaviors. Social support also plays a dual role: friends and families can encourage healthy behaviors like dietary changes but may also introduce challenges in social settings.

#### Coherence and care experiences

Some patients describe struggling to understand what their care entails, such as the purpose of specific medications, but that clear and accessible information helps them understand their treatment. Reliable information strengthens coherence, helping patients recognize the importance of treatment and self-management. While the volume of information can feel overwhelming, many patients describe taking an active role in gradually processing it over time. Trust in healthcare practitioners is central to this process; when information is delivered in a credible, up-to-date, and trustworthy manner, patients are better able to understand their condition and engage meaningfully in their care.

#### Timeline perceptions and care experiences

Access to information helps patients understand CKD as chronic while also reassuring them that treatments are available. Most patients understand that lifelong self-management is necessary, though some may view CKD as temporary or reversible, both of which may motivate active engagement. Trust in healthcare practitioners plays a central role in shaping patients' beliefs about disease duration and treatment across stages. Social support, particularly from family members at home, further helps patients cope with the thought of long-term CKD care and life on dialysis.

#### Emotional responses and care experiences

Emotional responses influence how patients experience their care, and these responses are in turn shaped by care experiences. Continuity of care reduces worry about disease progression, while disruptions in continuity can increase distress and undermine trust in healthcare. Access to reliable information helps reduce uncertainty, easing anxiety, while the demands of frequent visits, lifestyle changes, and heavy self-management responsibilities may lead to negative emotions such as frustration, or depression. At the same time, patients also describe experiencing positive emotions when reassured that treatments are available and that their own actions make a difference. Trust in competent healthcare practitioners further alleviates worry, whereas a lack of trust can exacerbate negative feelings. Social support plays a crucial role in helping patients cope with and process these emotional reactions.

## Discussion

The aims of this study were to explore CKD patients' illness perceptions and their experiences with care. We further aimed to identify how these illness perceptions and care experiences might interact to shape (motivation for) self-management behaviors.

Patients generally perceived CKD as a chronic but controllable condition with few noticeable symptoms and mild immediate consequences. While some patients described having experienced symptoms like high levels of fatigue, which impacts their ability to engage in different activities, current symptoms and consequences were mostly described as mild. Patients described that limited symptom experience and limited consequences reduced the presence of CKD in daily life, leading to not remembering or perceiving the need for self-management. This suggests that a low illness identity may reduce self-management behaviors, either through limited symptom experiences or not being able to attribute symptoms correctly, and aligns with some previous studies in CKD (Clarke et al., 2016; Meuleman et al., 2024).

Other studies have suggested that perceiving more symptoms, or attributing more symptoms to CKD (high illness identity) would, on the contrary, be associated with decreased adherence (Oliveira et al., 2023). This discrepancy could be explained by individual levels of self-efficacy. While perceiving health risks (such as symptoms) may motivate individuals to act to mitigate said risks, how and if they act is dependent on their self-efficacy to employ adaptive coping behaviors (Kok et al., 2018). How individuals react and behave as a result of experiencing symptoms or consequences of CKD could in other words be mediated by how confident they feel in their ability to self-manage those symptoms and CKD overall.

Although patients did not generally report severe or frequent immediate symptoms or consequences beyond regular clinic visits, potential future consequences were a common theme, with many expressing worry or uncertainty about disease progression and upcoming dialysis. This corresponds with the findings of Meuleman et al. (2024), who found that uncertainty about the future prevents patients from planning their future, which further increases concern. The findings of our study further suggest that patients may also be reluctant to plan for dialysis when they do not experience many symptoms. Low illness identities may thereby also act as a barrier for a smooth transition to dialysis, which has also been suggested by previous studies (Griva et al., 2020).

Most patients expressed confidence in their treatment and a sense of personal control, but uncertainty about the tangible effects of self-care behaviors sometimes diminished their motivation for self-management. Meuleman et al. (2024), also found that while patients viewed self-management as important, not perceiving its effects reduced their sense of control. Similarly, Muscat et al. (2021) reported that participants considered treatment essential for managing their illness, yet experienced growing concerns about its effectiveness as kidney function declined.

Greater illness understanding (coherence) was described as important for engagement in self-management, while gaps in understanding or misunderstanding of laboratory results and treatment mechanisms may decrease this engagement. Coherence was also described as closely related to emotional responses to CKD: These emotional responses—ranging from initial shock and fear to gradual adaptation—were primarily shaped by patients' perceived control, perceived and expected consequences, and understanding of CKD. Many patients described that their negative emotional responses were primarily alleviated by gaining more information and increasing their understanding of CKD and treatment. Negative responses (such as distress) have been linked to poor adherence in previous studies (e.g. Cardol et al., 2023) and is thereby an important factor to address.

Interactions between care experiences and illness perceptions revealed potential reciprocal influences that shape patients' engagement in self-management. Access to clear information, continuity of care, and social support seemed to help patients recognize and correctly attribute CKD symptoms, reinforcing illness identity and awareness of consequences. Continuity of care and trust in healthcare seemed to be related to reduced anxiety about disease progression, while disruptions in continuity or mistrust co-occurred with uncertainty and distress. Information and regular follow-up were described as factors which strengthen control beliefs by clarifying links between self-management and health outcomes, especially when patients do not perceive the effects of treatment or their actions. Coherence appeared to be increased through credible, comprehensible communication, enabling patients to understand CKD, and the purpose and value of treatment. Information and trust were also described as important for timeline perceptions, supporting acceptance of CKD as chronic while maintaining hope for improvement. Emotional responses were closely tied to care experiences: availability of reliable information and supportive practitioner relationships were described as reassuring, whereas heavy demands on patients to be active in their care and poor continuity increased worry and uncertainty. Overall, positive care experiences seem to foster adaptive illness perceptions and stronger self-management motivation.

Many of these findings align with previous evidence on treatment adherence and self-management in CKD. Interventions that enhance patients' knowledge and strengthen emotional self-management skills have been shown to improve adherence to treatment regimens (Farris et al., 2025; Peng et al., 2019). Research also demonstrates that stronger beliefs in the necessity of medication, paired with fewer concerns about potential harms, are associated with more consistent adherence behaviors (Sheils et al., 2024). In addition, meta-analyses indicate that behavior change strategies targeting knowledge, skills, behavioral regulation, social support, beliefs about consequences, and outcome expectations can effectively modify medication-related beliefs (e.g. perceived necessity, concerns about adverse effects) and improve a broad range of adherence outcomes (Ekholm et al., 2025; Sheils et al., 2024).

Taken together, these findings suggest that self-management could be promoted by improving illness perceptions and care experiences of patients with CKD. Applied strategies should specifically focus on correct attribution and meaning of symptoms (or lack thereof), consequences of CKD and impact of self-management behaviors, patient control beliefs and self-efficacy, patient understanding and knowledge about CKD and its treatment (coherence), building patient-practitioner trust through continuity of care, providing or facilitating psychosocial support, and facilitating relatable peer support. Suggestions on how healthcare practitioners could apply this in practice is outlined in Box 1.

Box 1. Recommendations for Practice. Based on these findings, healthcare practitioners should:

- Review lab results and CKD status with patients at each visit, to help them understand what their lab results mean, why symptoms may be minimal or absent, and how the disease may still progress despite the patient feeling well.
- Highlight how treatment and self-management contribute to slowing progression and maintaining quality of life, despite the patient not perceiving immediate effects.
- Provide clear, tailored education that fills knowledge gaps, including explanations of chronicity, treatment options, and self-management behaviors.
- Support patients in interpreting treatment and self-management effects by offering appraisal support so they can recognize the impact of their actions on their health.
- Validate patients' concerns and emotional reactions, while avoiding minimization of consequences. Encourage open discussion of fears, uncertainty, frustration, or other emotional reactions, and foster self-efficacy.
- Build trust through consistent, transparent communication and predictable follow-ups that help patients evaluate disease status and guide treatment decisions.
- Ensure continuity of care to reduce uncertainty and reinforce patient confidence in the healthcare system. Especially consider strategies or tools to improve perceived continuity in cases where patients cannot always see the same HCPs.
- Discuss environmental, social, and lifestyle factors that influence self-management (including work demands, caregiving responsibilities, financial barriers, and cultural context), and how patients can overcome barriers. Involve family members in ways that support the patient.
- Provide or coordinate psychosocial support, recognizing the emotional burden of frequent appointments, lifestyle changes, and the patients' responsibility of managing a chronic condition. Offer support for, for example, anxiety, frustration, or depression, and provide advice on accessing mental-health services.
- Facilitate access to meaningful social and peer support, acknowledging that patients differ in what is perceived as relatable or helpful. This could include matching patients with peers of similar age, CKD stage, or background, and recognizing that some may find peer or psychological support burdensome without adequate coordination.

A key strength of this study is its qualitative design, which offers rich insight into patient illness perceptions, the ways these perceptions shape self-management behaviors, and how both interact with care experiences. Quantitative approaches have dominated research on illness perceptions, leaving qualitative work in this area scarce (Meuleman et al., 2024), and our findings help capture nuances of patient experience that survey-based measures may miss. The attention to established trustworthiness criteria (Lincoln & Guba, 1985) further supports the credibility and transferability of the findings.

To our knowledge, this is the first study to qualitatively examine the relationship between illness perceptions, care experiences, and self-management among patients with CKD prior to kidney failure. By recruiting patients not yet on dialysis, including those at CKD stages 2 and 3, the study highlights how patients form understandings and strategies for managing CKD before reaching more advanced stages, offering valuable implications for early intervention and patient-centered care.

Despite the strengths of this study, several limitations should be acknowledged. The small sample size and the recruitment of patients from a single clinical setting limit the transferability of the findings, as care pathways and clinical practices may differ in other nephrology clinics.

A further limitation is related to the composition and selection of the sample. The sample consisted of patients in CKD stages 2 through 5, who can differ substantially in symptom burden, treatment intensity, and self-management demands. Combining them in the same analysis may thereby have obscured clinically meaningful differences in different stages of CKD. Selection effects may have shaped the sample further, as patients who were feeling more unwell may have been less willing or able to participate. The patterns that were identified as common, such as limited symptom experience, were supported by the interviews, but reflect the perspectives of the included patients. The results may thereby understate symptom burdens in the broader patient population. The sample size and analytic approach did not allow for systematic comparison of different stages of CKD and future research is needed to examine how perceptions and care experiences differ across CKD stages.

In addition, the post-encounter interview design meant that recruitment was tied to upcoming scheduled appointments at a single nephrology unit. This necessarily excluded patients who do not attend appointments or laboratory tests regularly, whose perspectives on CKD self-management may differ in important ways from those represented here. Purposive sampling was applied within this population to maximize variation in CKD stage, illness profile, educational background, and age, but the underlying sampling frame still shaped the range of experiences captured. It is also possible that an unusually positive or stressful recent encounter influenced how patients spoke about their broader CKD experience, although the protocol was structured to address broader questions first to mitigate this risk.

Finally, we did not include patients' causal beliefs about CKD in our interviews, limiting our ability to examine this component of illness perceptions. Although some patients spontaneously discussed potential causes, these accounts did not appear to relate to self-management in our sample. Future research could explicitly assess causal attributions to provide a more comprehensive understanding of how this might shape self-management.

## Conclusions

This study shows that CKD patients' illness perceptions and care experiences can jointly shape engagement in self-management behaviors. Limited symptoms, uncertainty about the effects of self-care, and emotional responses, such as worry about future progression often weakened motivation, while clear information, continuity of care, trust in healthcare practitioners, and supportive social networks strengthened patients' control beliefs, and understanding, and as a result, self-management. These findings align with previous evidence showing that improved knowledge, social support, and stronger treatment beliefs are linked to better adherence. Overall, enhancing patients' perceptions and care experiences may meaningfully promote self-management in CKD.

## Supplementary Material

Appendix II.pdf Appendix II.pdf

Supplementary material Appendix I.pdf

Supplementary material Appendix III.xlsx

## Data Availability

Individual-level data are not presented to ensure patient anonymity and confidentiality.
