# Supplementary material for: A qualitative investigation of experiences of care and illness perceptions related to self-management behaviors in chronic kidney disease
Source: Health Psychol Behav Med. 2026 Jul 8;14(1):2699481. doi: 10.1080/21642850.2026.2699481 (PMC13353484; doi:10.1080/21642850.2026.2699481)
Supplement: Appendix II.pdf [file RHPB_A_2699481_SM8457.pdf]

Interview script (translated from Finnish)

Intro:

- Briefly summarize research aims
- Emphasize that we are interested specifically about the respondents experiences
- Briefly explain confidentiality and anonymization
- Inform respondent about recorder and turn recorder on

Experiences and perceptions of CKD

I thought we would start by talking about you and your experiences of CKD, and then move on to talk about the encounter you just had with the healthcare staff. Does this sound good?

Would you like to start by telling me about your background and what brought you to the nephrology clinic?

- Supporting questions:
    - *When were you diagnosed?*
    - *What kind of thoughts did you have about getting diagnosed?*

Supporting questions for Illness Perceptions:

Consequences:
I do not have personal experience of CKD. How would you describe what CKD is and what kind of impact it has on your life to someone like me?

Identity/severity:
What kind of symptoms are associated with your condition?/What kind of symptoms of CKD have you experienced?

Treatment control
What kind of treatment have you received for your condition?
What kind of thoughts do you have about your treatment?
Do you feel that the treatment you are receiving is helping? In what way?

Personal control
Are there things you need to do or be mindful of because of CKD?
Do you feel like you can impact your health or your condition through your own actions? In what way?

Experiences of encounters with healthcare practitioners

You just had a meeting with the healthcare staff. Now I know where you have been and who you have met, but if you imagine that I know nothing about this encounter, how would you describe it to me?

Supporting questions
Who was present in the encounter?

What kind of topics did you discuss?
What kind of information did you receive about your care?
Did you talk about self-care?
Did you talk about dialysis?
Were you able to ask questions?
Was there something unclear, or something that came into your mind after the encounter?
How many times have you been to the nephrology clinic? What kind of encounters have you had before?
What will happen after this encounter? What kind of appointments are planned for the future?

Present respondent with think-out-loud assignment

Wrap up with
Is there anything else you would like to share about something we have or have not talked about?
Do you have any questions for me?
