# Supplementary material for: A qualitative investigation of experiences of care and illness perceptions related to self-management behaviors in chronic kidney disease
Source: Health Psychol Behav Med. 2026 Jul 8;14(1):2699481. doi: 10.1080/21642850.2026.2699481 (PMC13353484; doi:10.1080/21642850.2026.2699481)
Supplement: Supplementary material — Appendix I.pdf [file RHPB_A_2699481_SM8455.pdf]

**Typical patient journey from referral to kidney replacement therapy in the study context**

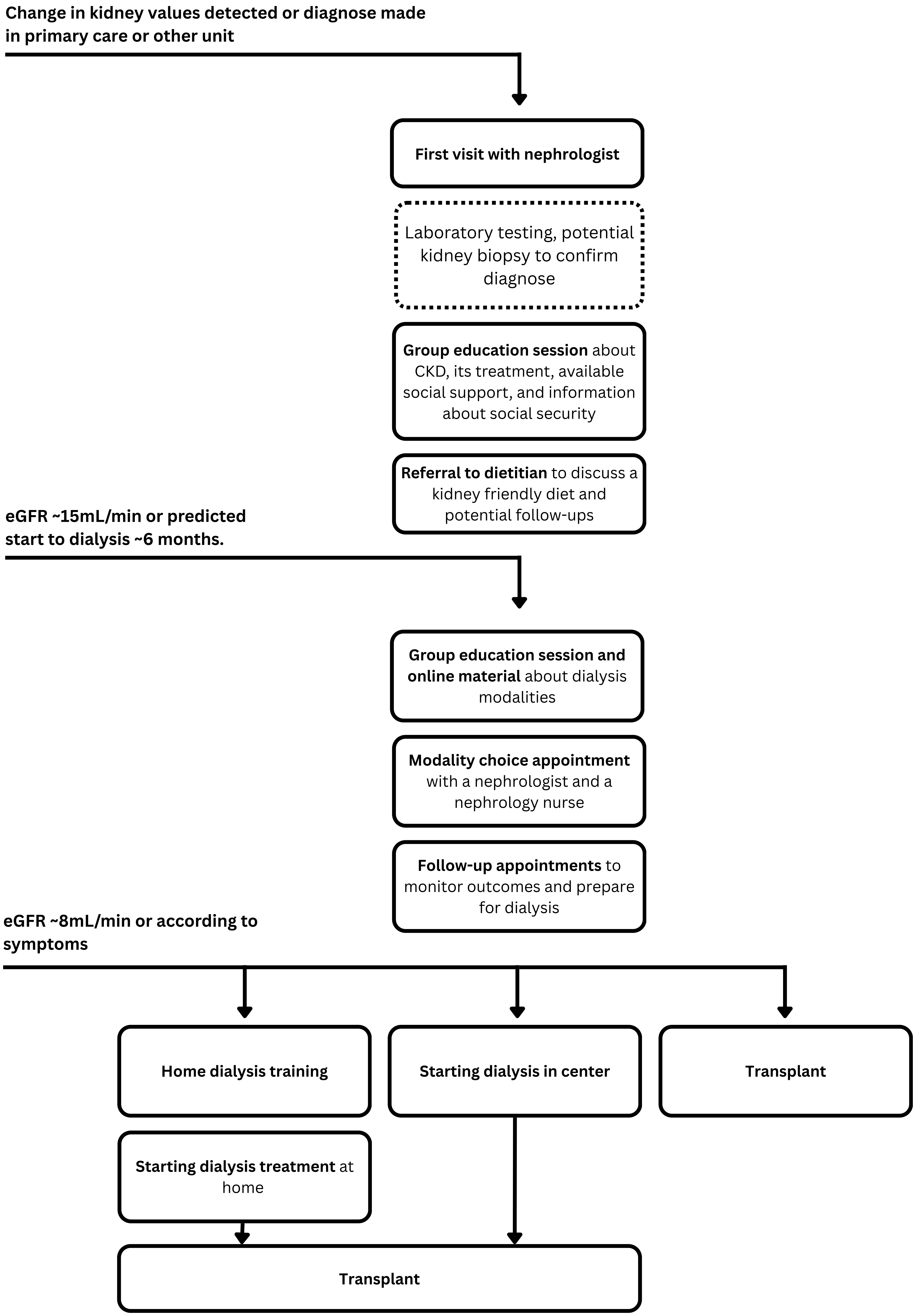


***Note.*** *Patients may also be referred to conservative care at any point along this trajectory. Individual journeys may vary in order and timing, not all patients attend every encounter shown, and some have additional appointments at other clinical units to address comorbidities.*
